# Childhood Chronic Physical Aggression Associates with Adult Cytokine Levels in Plasma

**DOI:** 10.1371/journal.pone.0069481

**Published:** 2013-07-26

**Authors:** Nadine Provençal, Matthew J. Suderman, Frank Vitaro, Moshe Szyf, Richard E. Tremblay

**Affiliations:** 1 Department of Pharmacology & Therapeutics, McGill University, Montreal, Quebec, Canada; 2 Research Unit on Children’s Psycho-Social Maladjustment and Ste-Justine Hospital Research Center, University of Montreal, Montreal, Canada; 3 Sackler Program for Epigenetics and Psychobiology, McGill University, Montreal, Quebec, Canada; 4 Department of Psychology and Pediatrics, University of Montreal, Montreal, Quebec, Canada; 5 School of Public Health, Physiotherapy and Population Science, University College Dublin, Dublin, Ireland; 6 McGill Centre for Bioinformatics, McGill University, Montreal, Quebec, Canada; 7 School of Psycho-Education, University of Montreal, Montréal, Quebec, Canada; The Nathan Kline Institute, United States of America

## Abstract

**Background:**

An increasing number of animal and human studies are indicating that inflammation is associated with behavioral disorders including aggression. This study investigates the association between chronic physical aggression during childhood and plasma cytokine levels in early adulthood.

**Methodology/Principal Findings:**

Two longitudinal studies were used to select males on a chronic physical aggression trajectory from childhood to adolescence (n = 7) and a control group from the same background (n = 25). Physical aggression was assessed yearly by teachers from childhood to adolescence and plasma levels of 10 inflammatory cytokines were assessed at age 26 and 28 years. Compared to the control group, males on a chronic physical aggression trajectory from childhood to adolescence had consistently lower plasma levels of five cytokines: lower pro-inflammatory interleukins IL-1α (*T*(28.7) = 3.48, *P = *0.002) and IL-6 (*T*(26.9) = 3.76, *P = *0.001), lower anti-inflammatory interleukin IL-4 (*T*(27.1) = 4.91, *P = *0.00004) and IL-10 (*T*(29.8) = 2.84, *P = *0.008) and lower chemokine IL-8 (*T*(26) = 3.69, *P = *0.001). The plasma levels of four cytokines accurately predicted aggressive and control group membership for all subjects.

**Conclusions/Significance:**

Physical aggression of boys during childhood is a strong predictor of reduced plasma levels of cytokines in early adulthood. The causal and physiological relations underlying this association should be further investigated since animal data suggest that some cytokines such as IL-6 and IL-1β play a causal role in aggression.

## Introduction

Physical violence is an important health problem, especially among males [1]. Longitudinal epidemiological studies show that physical aggression appears in early childhood, peaks between 2 and 4 years and decreases in frequency until early adulthood [2]–[4]. However, a small group of children (4–7%), mainly males, maintain a high level of physical aggression throughout childhood and adolescence [3]–[5]. These children tend to be impulsive, hyperactive, oppositional and rejected by their peers, they also tend to fail in school and have serious social adjustment problems during adulthood [5]–[9]. There is good evidence that the parents of children on a high trajectory of physical aggression had similar behavior problems and created early childhood family environments which did not support learning to regulate physically aggressive reactions [4], [10]–[14].

A growing body of research suggests that inflammatory cytokines might have systemic effects in addition to their traditional roles in the immune response. Recent studies have shown that cytokines are associated with various behavioral disorders such as anxiety, depression, suicide, childhood mood disorder and post-traumatic stress disorder (PTSD) [15]–[26]. It was also suggested that cytokines might play a role in the neurobiology of aggression since they are expressed in brain regions already known to be involved in aggression and behavior [27]–[29]. In humans, personality traits such as anger and hostility were found to be associated with increased levels of circulating IL-6 and C-reactive protein (CRP) [30]. Moreover, in non-chronically aggressive men, assessments of hostility, physical aggression, and verbal aggression were positively associated with lipopolysaccharide stimulated TNF-α expression in blood monocytes [31]. Moderate to severe maltreatment during childhood was also observed to be positively correlated with overall change in stress-induced IL-6 concentrations [32]. Neuroendocrine-immunological abnormalities that are established during a stressful childhood are thought to mediate the development of the pro-inflammatory phenotype in adulthood [33], [34]. Other studies examined the association between cytokines and aggression in animals. Gene knockout depletion of IL-6 (−/−) in mice resulted in increased aggression compared to control mice [35]. Moreover, in normal mice, over-expression of IL-6 in the brain increases affiliative behavior [35]. These data suggests that at least in mice IL-6 plays a causal role in aggression. Also in mice, IL-1β administration was found to reduce aggressive behavior in a dose-dependent manner supporting a causal relationship with aggression of this cytokine as well [36]. In mice bred for high aggression, IFNγ, IL-2 production, and T cell proliferation were higher than in mice bred for low aggression [37]. Interestingly, early life exposure to endotoxin, an immune activator, was found to reduce aggressive behavior in an aggressive strain of mice [38]. Taken together these studies suggest that cytokine expression profile may be a mediator linking experiences in early life to lifespan physical and mental health.

Although alterations in the levels of various cytokines have been shown to be associated with human disease and behavioral disorders, the association between cytokine levels and long-term regulation of physical aggression in humans is still unknown. We therefore used two longitudinal cohorts of children to examine whether cytokine levels in blood of young adults associate with their history of childhood physical aggression. To test this hypothesis, we measured an array of 10 inflammatory cytokines in a sample of males who had a history of chronic physical aggression between 6 and 15 years of age and compared them with boys from the same background who followed a normal physical aggression trajectory [2]. Our panel included a mix of five cytokines that were previously associated with human and animal behavioral problems and others whose association with these disorders was unknown.

## Results

### Plasma Cytokine Levels are Reduced in the Chronic Physical Aggression (CPA) Group

We first compared the two groups, controls and chronic physical aggressive (CPA) to identify possible confounders (Table 1). They did not differ significantly on age at the two blood samplings, on familial adversity during childhood, on their psychiatric record and on two behavior problems rated by teachers from kindergarten to high school: anxiety and inattention. As expected, a significantly higher proportion of the CPA group had a criminal record by age 24 and reported physical violence at age 21. Thus, the CPA group which was classified during childhood and adolescence maintained the phenotype into adulthood at the time when blood samples were collected. The CPA group also had a higher mean score for teacher rated hyperactivity and opposition between 6 and 15 years.

**Table 1:** Characteristics of the chronic physical aggression (CPA) group and control group (CG).

Variables	CG	CPA	Group
	Mean ± SD or % (n)	Mean ± SD or % (n)	
Age at ELISA time 1	25.64±2.08 (25)	25.28±2.75 (7)	*t*(30) = 0.37, *P = *0.71
Age at ELISA time 2	27.75±2.24 (16)	28.00±2.50 (5)	*t*(19) = −0.21, *P = *0.83
Familial adversity*	0.34±0.30 (25)	0.44±0.42 (7)	*t*(29) = −0.73, *P = *0.47
Psychiatric record (21 years)	33% (7/21)	43% (3/7)	*F* exact, 2 tailled: 0.67
Criminal record (21 years)	20% (5/25)	71% (5/7)	*F* exact, 2 tailled: *0.02*
Self reported violence (21 years)	10% (2/20)	57% (4/7)	*F* exact, 2 tailled: *0.02*
Hyperactivity (6 to 15 years)	1.20±0.96 (25)	2.43±1.27 (7)	*t*(30) = −2.79, *P = 0.009*
Oppositional disorder (6 to 15 years)	2.16±1.86 (25)	8.43±7.00 (7)	*t*(6.2) = −2.35, *P = *0.06
Anxiety (6 to 15 years)	3.36±1.93 (25)	3.43±1.51 (7)	*t*(30) = −0.09, *P = *0.93
Attention deficit (6 to 15 years)	3.20±2.22 (25)	4.00±2.00 (7)	*t*(30) = −0.86, *P = *0.40
C-reactive protein (mg/L)	0.51±0.57 (25)	0.83±0.92 (7)	*t*(30) = −1.14, *P = *0.26

* Include mother and father occupational score, familial status (monoparental vs biparental), mother and father age at birth of first child and the years of schooling of the mother and father.

We measured the plasma concentration of 10 cytokines using multiplex array-based ELISA at two time points (age 26 and 28) to obtain a reliable cytokine concentration for each subject. As expected, the cytokine concentrations at time 1 were significantly correlated with their concentrations 2 years later (*R* = 0.554, *P* = 1.48E-17). Using repeated measure ANOVA, no significant mean differences between times and no interactions with the aggression groups for all the 10 cytokines were revealed after correcting for multiple testing (Bonferoni correction with α ≤0.005). The analyses were therefore performed on the average cytokine concentration observed at time 1 and time 2.

The group differences were first assessed using a Student t-test and a multivariate test. They were further confirmed using Mann-Whitney and bootstrap non-parametric tests. We then performed linear regressions to determine whether the observed cytokine group differences were maintained when we adjusted for familial adversity and childhood hyperactivity.

**Figure 1:**
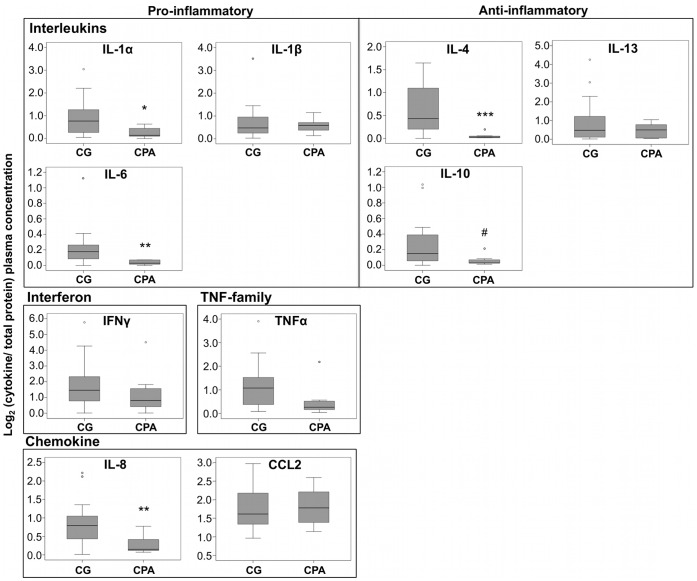
Decrease of IL-1α, IL-4, IL-6, IL-8 and IL-10 concentration in plasma is observed in the CPA group (n = 7) compare to the control group (n = 25). Log_2_ of the cytokine concentration normalized on the total amount of protein in plasma for each subject in each aggressive group is shown for 10 cytokines. Each boxplot represents the median (line), lower and upper quartiles (grey boxes), 95% confidence intervals (T-bars) and possible outliers (°) for each aggressive group per cytokine. Significant group difference was determined using student T-test with Bonferroni correction (α ≤0.005) and bootstrap (see methods). CPA indicates the chronic physical aggression trajectory group and CG the control group. MANOVA combining all 10 cytokines: *F*(10) = 2.9, *P* = 0.019. *** *P*≤0.0001, ** *P*≤0.001, * *P*≤0.005, # *P*≤0.01 from Student T-test (two-tailed).

Results obtained from the multiplex ELISA for 10 cytokines concentration in plasma analyzed for the CPA group and control group are displayed in Figure 1. There was a multivariate effect of the aggression group (*F*(10) = 2.9, *P = *0.019). Student T-test analysis with Bonferroni correction (α ≤0.005) showed that the CPA group compared to the control group had lower pro-inflammatory interleukins: IL-1α (*T*(28.7) = 3.48, *P = *0.002) and IL-6 (*T*(26.9) = 3.76, *P = *0.001); lower chemokine: IL-8 (*T*(26) = 3.69, *P = *0.001); and lower anti-inflammatory interleukin: IL-4 (*T*(27.1) = 4.91, *P = *0.00004) concentration. A trend was also observed for the other anti-inflammatory interleukin interrogated, IL-10 (*T*(29.8) = 2.84, *P = *0.008), going in the same direction with lower concentration in the CPA group. There were no other significant differences between the groups for the other cytokines analyzed (IL-1β (*T*(30) = 0.38, *P* = 0.71), IL-13 (*T*(30) = 1.08, *P* = 0.29), CCL2 (*T*(30) = −1.66, *P* = 0.87), IFNγ (*T*(30) = 0.75, *P* = 0.46) and TNF-α (*T*(30) = 1.46, *P* = 0.15)). Interestingly, Levene’s test of equality of variances indicated higher variance in basal cytokine concentration for IL-1α, IL-4, IL-6, IL-8 and IL-10 for the control group compared to the CPA group. These results show a tighter and lower basal concentration of pro and anti-inflammatory cytokines in the CPA group.

To further test the hypothesis that cytokines are associated with CPA rather than a confounder, we conducted regression analyses using linear mixed model on each cytokine adjusting for familial adversity. Familial adversity was chosen since it is known to predict CPA trajectory membership [4] as well as immune response deficits [39]. Even with our small samples size, the CPA group was still significantly associated with lower IL-1α (*P* = 0.0004), IL-4 (*P*<0.0001), IL-6 (*P* = 0.0005) and IL-8 (*P* = 0.001) levels and marginally associated with IL-10 (*P* = 0.008) levels after Bonferroni correction (α <0.005). We then tested the association between cytokine levels and childhood hyperactivity as a possible confounder since the CPA group had a higher hyperactivity average score than the control group and hyperactivity is associated with CPA from childhood to adolescence [40]. With or without adjusting for familial adversity, none of the 10 cytokines significantly associated with childhood hyperactivity at *P*<0.05. Adjusting for both, family adversity and childhood hyperactivity in our regression analysis showed that the CPA group was still significantly associated with lower level of IL-4 (*P* = 0.023) and IL-8 (*P* = 0.017) and marginally associated with lower level of IL-1α (*P* = 0.060), IL-6 (*P* = 0.082) and TNF-α (*P* = 0.091). In addition, cytokine levels may vary in response to external stimuli such as infections. CRP, a well-known marker of infection, was also shown to associate with the hostility trait in humans [30]. In our samples, CRP levels in plasma were not different between CPA and control groups (Table 1). Adjusting for CRP levels in addition to family adversity and hyperactivity in our regression analysis showed that the CPA group was still significantly associated with lower level of IL-4 (*P* = 0.025) and IL-8 (*P* = 0.019) and marginally associated with lower level of IL-1α (*P* = 0.062), IL-6 (*P* = 0.088), IFNγ (*P* = 0.099) and TNF-α (*P* = 0.098).

### Cytokine Level Measures Classify the Two Aggression Groups

We used logistic regression to test how well the cytokine concentrations could classify the subjects in the two aggression groups (CPA-CG). Two cytokines were sufficient to classify all but 5 subjects in the appropriate aggression group. Cytokine pairs consisted of IL-4 and either CCL2, IL-13, IL-8 or IL-1β. All misclassifications were eliminated when we included 4 cytokines: IL-1α combined with two of the four other differentiating cytokines (IL-6/IL-4/IL-10/IL-8) and with one non-differentiating cytokine (IL-1β or CCL2). According to the Akaike Information Criterion (AIC), these 4-cytokine classifiers were the strongest of all possible cytokine classifiers.

### Secondary Analysis

As the sample number was small and there was unequal variance in our sample we also applied Mann-Whitney and bootstrap nonparametric tests. Mann-Whitney analyses for each of the cytokines show similar results to the one obtained by Student t-test with significant group difference at α ≤0.05 seen for IL-1α (*P* = 0.036), IL-4 (*P* = 0.002), IL-6 (*P* = 0.007), IL-8 (*P* = 0.010). Bootstrap analysis done on 1000 permutations found the same cytokines to be associated with the aggression groups (IL-1α (*P = *0.011), IL-4 (*P = *0.002), IL-6 (*P = *0.018), IL-8 (*P = *0.006) and IL-10 (*P = *0.036)).

## Discussion

Several lines of evidence suggest that cytokines are associated with animal and human aggression [27]–[29]. Our analyses of data over a 22 year period show for the first time that there is a long term association between cytokine levels in plasma and chronic physical aggression: young adult men with a history of chronic physical aggression during childhood have lower baseline concentrations of three pro (IL-1α, IL-6 and IL-8) and two anti (IL-4 and IL-10) -inflammatory cytokines than comparable young adult men without a history of chronic physical aggression during childhood. Remarkably, this strong association was found although chronic physical aggression was assessed by teachers during childhood and the cytokines were measured from blood samples when the subjects were 26 and 28 years. Four of the ten cytokines analyzed accurately classify all the subjects in the aggressive and non-aggressive groups.

The mechanisms responsible for the low cytokine levels in the chronic aggression group or their possible role in aggression still need to be determined in future experiments. However, several molecules previously shown to be involved in aggression could either regulate cytokine levels in brain and plasma or be regulated by cytokines. First, high cortisol levels were found to be associated with high levels of aggression in adolescent males from the same sample [41]. Cortisol levels are known to regulate immune and inflammatory responses [42]. Second, Vasopressin, a mediator of the HPA axis activity released in the brain enhances arousal and aggression [43]. Brain vasopressin is also involved in stress-induced suppression of immune functions in rats [44], [45]. Third, serotonin, a key player in aggressive behavior, is induced by cytokines, such as IL-6 and IL-1β, in brain and in blood [46]–[48]. Serotonin is also known to be involved in regulating IL-4, IL-8, IL-6, TNF-α and IL-1 expression and secretion through the CREB signaling pathway [49], [50]. Together, these studies suggest a link between known mediators previously shown to be involved in aggression and cytokines.

The main remaining question is causality. Does chronic aggression during childhood result in lowered cytokine activity or does lowered cytokine activity result in more aggression? Defining causal relationships in human studies is extremely difficult. However, animal studies where causality could be experimentally tested have shown a causal relationship between levels of one of the cytokines examined here IL-6 and aggression. Gene knockout depletion of IL-6 (−/−) in mice resulted in increased aggression compared to control mice, which is consistent with our data showing reduced IL-6 in the CPA group [35]. We don’t know whether these results in mice could be translated to humans. However, the associations observed in our study taken together with the rodent results are consistent with the hypothesis that cytokines might play a role in human chronic physical aggression.

The main limitation of the present study is the small sample size of the chronic aggression group. The two longitudinal studies we used to recruit subjects had followed more than 1000 males from childhood to adolescence. Unfortunately, young adult Caucasian males with a history of chronic physical aggression during childhood are relatively rare [5] and difficult to recruit for biological sampling over a two year period. Thus, replications of the present study with other longitudinal samples are obviously needed. The replications we have done with the Mann-Whitney and bootstrap nonparametric tests indicate that the observed significant differences between the two groups are robust. Nonetheless, the small sample size prevented the introduction of many confounders into the analyses. We did adjust for one of the most likely confounder, family adversity. Childhood family adversity is a well known risk factor for chronic physical aggression [4] as well as immune response deficits [39]. Even with our small samples size, the significant group differences for cytokine levels were maintained when we adjusted for childhood family adversity in the regression analysis. As expected the two groups were also significantly different on other variables that are known to be strongly associated with chronic physical aggression trajectories from childhood to adolescence: childhood hyperactivity, adolescence physical violence and adulthood criminal behavior (Table 1) [2], [5]. Although cytokine levels have been shown to associate with psychiatric diseases such as major depression [51] the two groups of males were not significantly different on levels of anxiety and presence of psychiatric diagnoses (Table 1). We also determined whether physical health problems could explain the cytokine level differences between the two groups. Two members of the control group had cardiovascular disease and two others had respiratory disease. Excluding these subjects from our analysis did not change the significant cytokine differences observed between the two groups. We quantified CRP levels, a well-known marker of infection, and found no differences between CPA and control groups (Table 1). Because our small sample size prevents the use of many confounders, we attempted to control for the three main confounders; family adversity, hyperactivity and CRP levels. Results showed that the CPA group was still significantly associated with lower level of two cytokines (IL-4 and IL-8). There were no differences in age between the groups and no significant correlations were found between age and cytokine levels. Taken together, these results suggest that chronic physical aggression during childhood is a predictor of cytokine levels during early adulthood.

Diurnal variation has been reported for IL-6 [52], TNF-α [53], IL-4 [54], IL-13 [55], IFNγ, IL-10 and IL-1 [56]. In general, their levels peak at night and/or early morning. To account for theses variations, all the blood samples were taken during daytime between 13∶00 and 20∶00. Future studies are needed to determine whether similar results would be obtained for IL-1α, IL-4, IL-6, IL-8 and IL-10 when samples are taken at different time points during the day. However, the relatively high correlation between samples at 26 and 28 years (*R* = 0.554, *P* = 1.48E-17) suggests that one daytime sample is a relatively robust assessment.

### Conclusions

This study has several implications. The results suggest that cytokines may be involved in chronic physical aggression, hence that a peripheral immune component may play a key role in regulating these behavioral states. We also showed that measuring the levels of a panel of 4 cytokines in plasma could accurately discriminate adult males with and without a history of childhood chronic physical aggression. This raises the possibility that cytokines could become peripheral biomarkers of risk for chronic physical aggression and related serious behavioral problems such as hyperactivity. New longitudinal studies that repeatedly assess cytokine, cortisol and physical aggression from early childhood onwards are needed to define the temporal relationship between changes in basal cytokine levels, cortisol and appearance of aggressive behaviors.

## Materials and Methods

### Participants

The subjects were recruited from participants in two longitudinal studies of child development [13], [57]. We recruited two groups of Caucasian males who were born in families with a low socioeconomic status and were living at the time of the present study within 200 km from our laboratory. The first group had a history of chronic physical aggression from age 6 to 15 years (chronic physical aggression group, CPA). The second group was recruited from the same longitudinal studies but included only those who did not have a history of chronic physical aggression from age 6 to 15 (Control group, CG).

A total of 65 eligible subjects accepted to participate (8 CPA and 57 CG). One of the 8 CPA subjects had to be discarded because of data quality and for economic reasons we randomly reduced the CG group to 25. Characteristics of the 2 groups are presented in Table 1.

### Ethics Statement

After complete description of the study to the subject, all participants provided written informed consent. The study was carried out in accordance with the Declaration of Helsinki, and was approved by the research ethics committee of the University of Montreal pediatric hospital (St-Justine Hospital).

### Plasma Isolation and ELISA

Blood samples (8 to 10 ml) were drawn in EDTA coated-tubes and shipped on ice to the laboratory within 24 hours. Blood samples were immediately resuspended in equal volume of PBS (1X) and plasma isolation was performed by centrifugation on a Ficoll-Paque gradient (GE healthcare). The samples were then frozen and stored at −80°C in aliquots of 1 ml. Quantification of 10 cytokines in plasma was done by ELISA using the Quantibody® Human Inflammation Array 1 from RayBiotech following the manufacturer instructions for both time points [58]. Briefly, 100 µl of plasma were diluted 3 times in PBS (1X) and incubated at 4°C overnight with the arrayed antibody supports. Each array was composed of 16 wells, 5 wells were used for cytokine standard dilutions, one for the negative control (PBS) and the remaining 10 for the plasma samples. The second incubation consisted of adding a cocktail of biotinylated antibodies for 1 hour and the third incubation with Alexa Fluor 555-conjugated streptavidin was performed in the dark for 1 hour at room temperature. Samples were washed 5 times with buffer I and two times with buffer II following each incubations. Arrays were then scanned with the Agilent C-scanner (excitation: 555 nm, emission: 565 nm and resolution: 10 µm) and data extraction was done using ArrayVision 8.0. Each array consisted of quadruplicate quantification of each cytokine per sample and standard. Absolute concentrations for each cytokine were calculated from the standard curve with the Q Analyser software (RayBiotech). Repeat measurements were done for 8 samples to validate the results at a 2 year interval (26 to 28 y). All cytokine concentrations included in the study were within their standard curve ranges, one CPA subject was not included in the study since his concentration was outside the expected range of the standard curve. Only 4 subjects had undetectable levels (<0.2) for at least one cytokine either at time 1 or time 2 (1 CPA for IL-1α, 1 CG for IL-4, 1 CPA and 1 CG for IL-6). The cytokine concentrations were normalized to total protein plasma concentrations, which were determined using a standard Bradford assay.

C-reactive protein levels in plasma were quantified by Bio-Medic Laboratories using a particle enhanced immunoturbidimetric assay. Briefly, human CRP agglutinates with latex particles coated with monoclonal anti-CRP antibodies. The precipitate causes an increase in the intensity of scattered light and is proportional to the amount of CRP in the sample. All values were below the reference value for plasma (≤10.00 mg/L).

### Assessment of Subjects’ Familial Adversity, Behavior Problems, Psychiatric Diagnoses and Criminal Records

#### Familial adversity

The index of family adversity is a composite score of the degree of adversity in families ranging from 0 to 1 which has been used regularly with these cohorts. The index includes parent’s level of education, type of employment, age at the birth of their first child and marital status when the subjects were age 6 [14], [59].

#### Physical aggression and other behavior problems

In the course of the longitudinal studies, teachers annually rated the frequency of boys’ physical aggression, opposition, hyperactivity, inattention and anxiety from kindergarten to secondary school with the Social Behavior Questionnaire [59]. The physical aggression ratings were used to trace the developmental trajectories and create the CPA group and the control group (see Broidy et al., 2003 and Nagin & Tremblay, 1999 for details of the trajectory analyses). *Self reported violence.* During the data collection at 21 years, subjects were asked how often in the past year they had been implicated in physical fights and how often they had physically attacked someone.

#### Criminal record

Canadian youth between 13 and 17 years who commit delinquent acts are referred to the juvenile courts. From official records we identified subjects who were found guilty by a juvenile court. From official records we also identified subjects in each group who had been convicted of a criminal offence between 18 and 24 years.

#### Psychiatric disorders

When the subjects were 15 years structured psychiatric interviews were used with the mothers and the subjects to assess the following psychiatric disorders: simple phobia, anxiety of separation, generalized anxiety, hyper anxiety, major depression, dysthymia, oppositional disorder and conduct disorder [60]. Subjects were also asked if they had a psychiatric record during the interview at 21 years.

### Statistical Analysis

The normalized cytokine concentrations and CRP values in plasma showed a right-skewed distribution. In order to achieve normality, the data were transformed using log based 2. The correlation between cytokine concentrations at time 1 and time 2 was calculated using linear mixed effects model with concentrations grouped by subject and cytokine in the R software environment for statistical computing [61]. The normality tests, repeated measures and multivariate ANOVAs, Student t-test (two-tailed) and Mann-Withney analysis were done using SPSS statistic 19.0 (IBM). Bootstrap was performed using SPSS statistic 20.0 (IBM). For the Student t-test, P-value for unequal variance was used when Levene’s test of equality variances was significant at *P*<0.1. Linear regression analyses were performed using statistical analyses software (SAS 9) version 9.2 (SAS Institute, Cary, NC). The cytokine classifications were assessed using logistic regressions and the Akaike Information Criterion (AIC) values in R software environment for statistical computing.

Excluding the 4 undetectable values, mentioned above, in the final analysis did not affect the significant group differences observed for all the cytokines except for IL-10. IL-10 group difference observed without the undetectable values gives a slightly lower P value (*P* = 0.004) than by including them (*P* = 0.008). Since for 9 out of 10 cytokines the results were not affected, the analysis presented here included these 4 values of 0 in all the analysis to allow equal number of subject for each cytokine. Nine of the 10 cytokines analyzed (excluding CCL-2) had at least one or more values outside of the distribution (> upper quartile +1.5 times the interquartile range (IQ)) that could be considered as possible outliers. Comparing the groups with these data brought into range (equal to upper quartile +1.5*IQ) [62] did not change the results. Therefore, outliers are not of an issue for the analyses.
